# Aortic disease and cardiomyopathy in patients with a novel *DNMT3A* gene variant causing Tatton-Brown–Rahman syndrome

**DOI:** 10.1186/s13148-024-01686-y

**Published:** 2024-06-06

**Authors:** Dovile Zebrauskiene, Egle Sadauskiene, Justas Dapkunas, Visvaldas Kairys, Joris Balciunas, Aleksandras Konovalovas, Ruta Masiuliene, Gunda Petraityte, Nomeda Valeviciene, Mindaugas Mataciunas, Jurate Barysiene, Violeta Mikstiene, Migle Tomkuviene, Egle Preiksaitiene

**Affiliations:** 1 Department of Human and Medical Genetics, Institute of Biomedical Sciences, Faculty of Medicine, Vilnius University, Santariskiu 2, 08661 Vilnius, Lithuania; 2 Clinic of Cardiac and Vascular Diseases, Institute of Clinical Medicine, Faculty of Medicine, Vilnius University, Vilnius, Lithuania; 3 Department of Bioinformatics, Institute of Biotechnology, Life Sciences Center, Vilnius University, Vilnius, Lithuania; 4 Department of Biological DNA Modification, Institute of Biotechnology, Life Sciences Center, Vilnius University, Sauletekio 7, 10257 Vilnius, Lithuania; 5 Institute of Biosciences, Life Sciences Center, Vilnius University, Vilnius, Lithuania; 6 Faculty of Medicine, Vilnius University, Vilnius, Lithuania; 7 Department of Radiology, Nuclear Medicine and Medical Physics, Institute of Biomedical Sciences, Vilnius University Faculty of Medicine, Vilnius, Lithuania

**Keywords:** Tatton-Brown–Rahman syndrome, *DNMT3A*, DNA methylation, Transcription, Aortic dilatation, Cardiomyopathy, Mitral valve, Arrhythmias

## Abstract

Tatton-Brown–Rahman syndrome (TBRS) is a rare congenital genetic disorder caused by autosomal dominant pathogenic variants in the DNA methyltransferase *DNMT3A* gene. Typical TBRS clinical features are overgrowth, intellectual disability, and minor facial anomalies. However, since the syndrome was first described in 2014, a widening spectrum of abnormalities is being described. Cardiovascular abnormalities are less commonly reported but can be a major complication of the syndrome. This article describes a family of three individuals diagnosed with TBRS in adulthood and highlights the variable expression of cardiovascular features. A 34-year-old proband presented with progressive aortic dilatation, mitral valve (MV) regurgitation, left ventricular (LV) dilatation, and ventricular arrhythmias. The affected family members (mother and brother) were diagnosed with MV regurgitation, LV dilatation, and arrhythmias. Exome sequencing and computational protein analysis suggested that the novel familial DNMT3A mutation Ser775Tyr is located in the methyltransferase domain, however, distant from the active site or DNA-binding loops. Nevertheless, this bulky substitution may have a significant effect on DNMT3A protein structure, dynamics, and function. Analysis of peripheral blood cfDNA and transcriptome showed shortened mononucleosome fragments and altered gene expression in a number of genes related to cardiovascular health and of yet undescribed function, including several lncRNAs. This highlights the importance of epigenetic regulation by DNMT3A on cardiovascular system development and function. From the clinical perspective, we suggest that new patients diagnosed with congenital DNMT3A variants and TBRS require close examination and follow-up for aortic dilatation and valvular disease because these conditions can progress rapidly. Moreover, personalized treatments, based on the specific DNMT3A variants and the different pathways of their function loss, can be envisioned in the future.

**Supplementary Information:**

The online version contains supplementary material available at 10.1186/s13148-024-01686-y.

## Introduction

Tatton-Brown–Rahman syndrome (TBRS) is an autosomal dominant overgrowth syndrome caused by heterozygous and usually de novo pathogenic variants of the DNA methyltransferase *DNMT3A* gene [1]. The clinical features of the syndrome partly overlap with those of other overgrowth syndromes, such as Sotos syndrome, *EZH2*-related Weaver syndrome, and a few others [2]. TBRS was first described in 2014, following a report of 13 individuals with de novo heterozygous *DNMT3A* variants [3]. Among these, two were adults and the others were children. A subsequent follow-up study analyzed 55 individuals with de novo* DNMT3A* variants, including 13 previously reported individuals [1]. Of this cohort, 15 were adults. To date, most individuals with TBRS reported in the literature are children. Affected individuals typically share a phenotype of overgrowth, intellectual disability, and minor facial anomalies [1, 4]. Subtle dysmorphic features, such as a round face with coarse features, horizontal thick eyebrows, prominent upper central incisors, and narrow palpebral fissures, are the most easily recognizable in adolescence and less specific in early childhood or at adult age [1]. Other frequent clinical features include joint hypermobility, obesity, hypotonia, behavioral and psychiatric issues, kyphoscoliosis, and afebrile seizures [1]. TBRS could be linked to an increased risk of acute myeloid leukemia development [1, 5]. An increasingly wider spectrum of neurological, skeletal, and cardiological abnormalities have been found in adults [1, 5]. Cardiovascular abnormalities are less commonly reported, including patent foramen ovale and ductus arteriosus, septal defects, mitral and tricuspid regurgitation usually identified early in life, cardiomyopathy, and aortic root dilatation—in adults [1, 4–10]. The association between TBRS and aortic root dilatation remains less well-defined. Cardiovascular anomalies can be life threatening, although to date, no sudden cardiac death and aortic dissection in patients with TBRS have been reported.

**Fig. 1:**
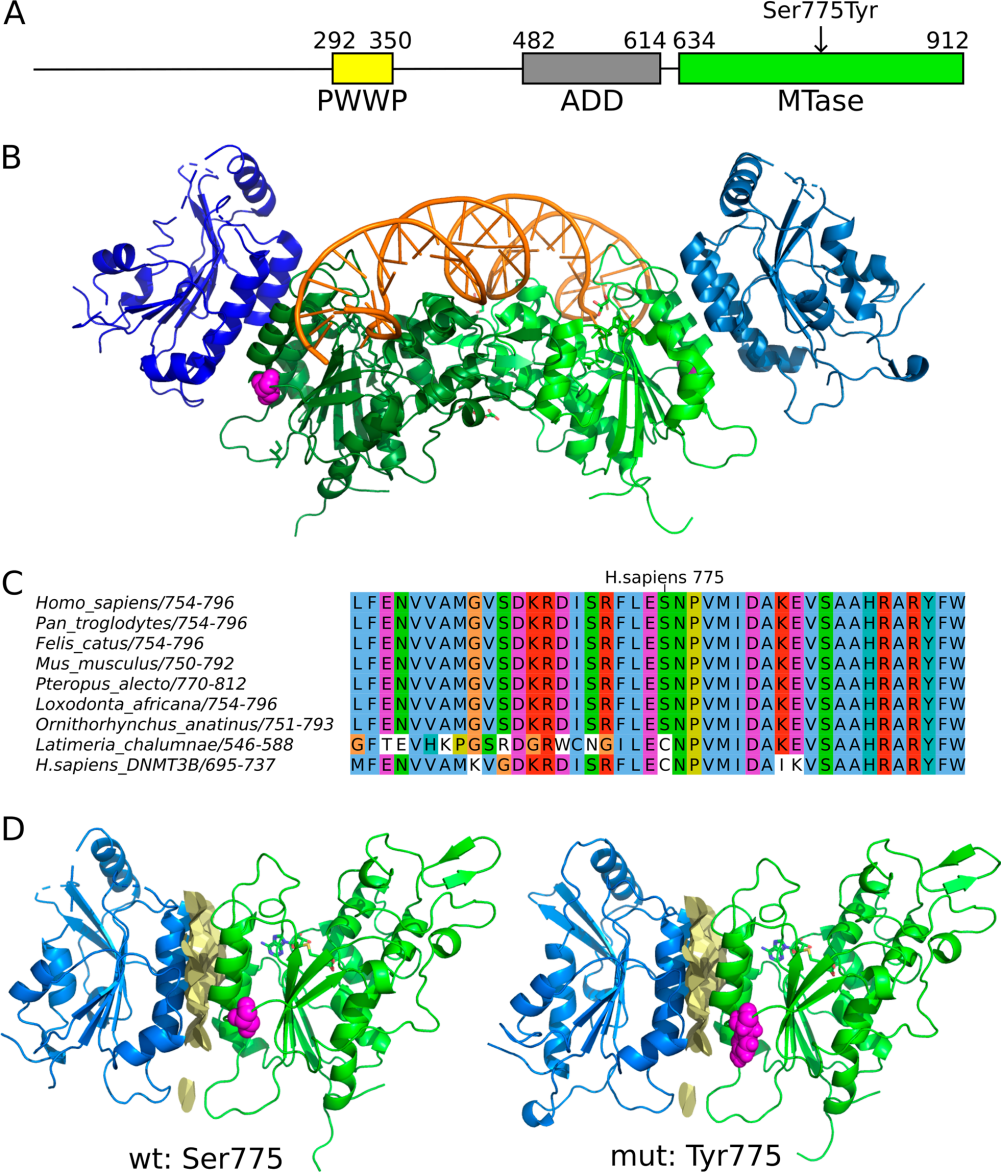
Analysis of DNMT3A sequence and structure. **A** Domain organization of DNMT3A. Yellow: Proline-tryptophan-tryptophan-proline (PWWP) domain, gray: ATRX-DNMT3A-DNMT3L-type zinc finger (ADD) domain, green: C5-type methyltransferase (MTase) domain. **B** Structure of DNMT3A in complex with DNMT3L and DNA (PDB: 5YX2). DNMT3A is in green, DNMT3L is in blue, DNA is in orange, and the Ser775 residue is shown in magenta colored spheres. **C** Multiple sequence alignment of representative mammalian DNMT3A proteins, Latimeria DNMT3A and human DNMT3B, showing the conserved region containing the Ser775 residue. **D** The mutated residue 775 is positioned near the interface of interaction between DNMT3A and DNMT3L (PDB: 5YX2). Interface is shown in yellow

DNMT3A is a conserved multidomain protein involved in an intricate network of interactions. DNMT3A is a DNA (cytosine-5)-methyltransferase that is involved in the formation and maintenance of genomic DNA methylation, which is one of the most important epigenetic processes [11, 12]. DNA methylation plays a key role in modulating gene expression, genomic imprinting, X-chromosome inactivation, and has been implicated in tumorigenesis [13–15]. Studies of methylation in mouse models and humans have revealed that pathogenic TBRS variants in *DNMT3A* lead to a distinct hypomethylation pattern. This finding implies that such variants induce a loss-of-function effect on methyltransferase, potentially leading to broad dysregulation of gene expression [16]. DNMT3A has three functional domains: a proline-tryptophan-tryptophan-proline (PWWP) domain, an ATRX-DNMT3A-DNMT3L-type zinc finger (ADD) domain and a C-terminal DNA methyltransferase domain (Fig. 1A) [3]. The clustering of reported pathogenic missense variants of DNMT3A within these three functional domains suggests that these variants are likely to cause disease by disrupting domain activity [1, 8]. The similarity in hypomethylation patterns between pathogenic missense variants and loss-of-function variants lends support to this hypothesis [16]. Analysis of the DNMT3A protein showed that the C-terminal domain adopts the typical cofactor AdoMet-dependent methyltransferase (MTase) fold with an active DNA methylation site, an AdoMet-binding pocket, and DNA-binding loops. The regulatory PWWP and ADD domains determine the activity of the MTase domain and target it to specific chromatin regions by interacting with the histone epigenetic code [17]. The ADD domain exerts an auto-inhibitory effect that is lifted by substantial structural reorganization upon interaction with a modification-free histone H3 tail [18]. DNMT3A activity is dependent on autodimerization and interactions with DNMT3L or DNMT3B3 that are catalytically inactive yet essential regulatory paralogs (Fig. 1B) [19, 20] that bind to the DNMT3A MTase domain, stabilizes it, and read the histone code [21–23].

Recent studies suggest that DNA methylation has an important role in cardiomyocyte homeostasis, cardiac stress responses and progression of cardiac fibrosis. Knockout of DNMT3A in human induced pluripotent stem cell-derived cardiomyocytes resulted in changes of cardiomyocyte morphology and function: altered contraction kinetics, intracellular lipid accumulation, impaired glucose metabolism and lower glycolytic enzyme expression [24]. In other studies, clonal hematopoiesis-driver *DNMT3A* mutations were associated with increased cardiovascular risk: the development of atherosclerosis, inflammation, progression and poor prognosis of chronic heart failure [25–28].

In this report, we describe a two-generation family of three affected adult individuals diagnosed with TBRS and Ser775Tyr missense mutation in the DNMT3A MTase domain. In detail, we highlight the variable manifestation of multiple cardiovascular deviations and present analysis of peripheral blood transcriptome and cfDNA for deeper understanding of the underlying molecular pathways.

## Materials and methods

### Clinical evaluation

**Fig. 2:**
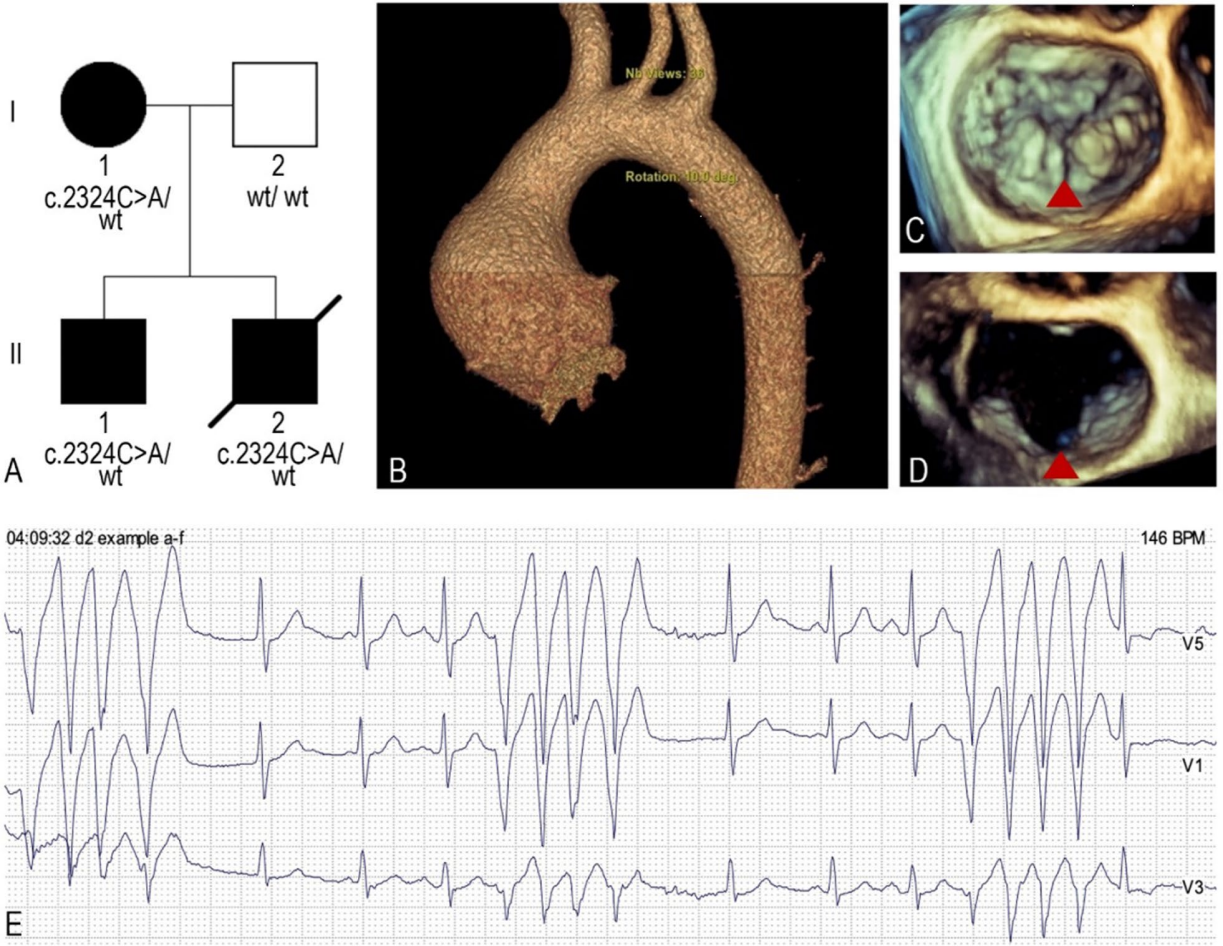
**A** Genealogy of the family: II-2—proband (patient 1), II-1—affected sibling (patient 2), I-1—mother. **B** Proximal ascending aorta aneurysm (6 cm) on computed tomography. **C**, **D** Cleft posterior mitral valve leaflet (red triangle) between P2 and P3 segments on transesophageal echocardiography. **E** Short episodes of wide QRS complex tachycardia corresponding to non-sustain ventricular tachycardia during the 24-h Holter monitoring of the proband

We report three affected individuals in the family: two men aged 34 years (proband) and 39 years (affected sibling), and their mother, aged 58 years (Fig. 2A). The father of the siblings is healthy. The maternal grandfather was known to have had pectus excavatum.

Patient 1 (Decipher 524,710). The proband, a 34-year-old male, was referred to a cardiologist following episodes of weakness, dizziness, cold sweats, heart palpitations, and dyspnea on exertion (Table 1, proband). These symptoms persisted for several years without progression. Transthoracic echocardiography (TTE) revealed a left ventricular (LV) and aortic root dilatation (left ventricular end-diastolic diameter (LVEDD)—6.9 cm; indexed 2.79 cm/mm^2^), moderate mitral regurgitation, and preserved LV ejection fraction (EF). Chest computed tomography (CT) angiography confirmed aortic root dilatation (4.8 cm at the sinuses, 4.1 cm—proximal ascending aorta). Cardiac magnetic resonance imaging (MRI) additionally showed signs of LV non-compaction, LV dilatation (LVEDD—6.9 cm), and no late gadolinium enhancement (LGE). 24-h Holter monitoring revealed a short episode of non-sustained ventricular tachycardia (five QRS complexes with a heart rate of 189 bpm) and intermittent second-degree type I atrioventricular (AV) block.

**Table 1:** Descriptive analysis of TBRS-affected family members

	Proband	Affected sibling	Mother
Sex	Male	Male	Female
Age at diagnosis	34 years	39 years	58 years
Height	191 cm	192 cm	169 cm
Weight	120 kg	140 kg	130 kg
BMI	32.89	37.98	44.98
Facial appearance	Head circumference 63 cm, hypertrichosis, coarse facial features, synophrys, improper growth of teeth, fractured front teeth, hypertelorism	Head circumference—62 cm (90–97‰), synophrys, milder facial features, horizontal thick eyebrows, abdominal stretch marks	Head circumference 56 cm, milder facial features, horizontal thick eyebrows
Intellectual disability	Verbal IQ score of 75—poor verbal comprehension; non-verbal IQ score of 55—mild intellectual disability, special education in a secondary school	Attended mainstream school following the standard curriculum	Attended mainstream school following the standard curriculum
Skeletal deformity	Kyphoscoliosis; pectus excavatum surgically corrected in childhood	Scheuermann’s kyphosis with critical spinal cord compression, surgically corrected at age 38	Spinal osteochondrosis, spondyloarthrosis
LV dilatation (LVEDD) on TTE	6.9 cm	5.9 cm	6.7 cm
Aortic root dilatation	Proximal ascending aorta aneurysm (6 cm)	Aortic sinus diameter—4.5 cm	–
MV/TV incompetence	Cleft posterior MV leaflet, mitral prolapse, severe (grade 3) MV regurgitation	Mild (grade 1) MV regurgitation	Mild-to-moderate (grade 1–2) MV regurgitation; mild (grade 1) TV regurgitation
LV non-compaction	+	−	−
Arrhythmias	Non-sustained ventricular tachycardia, second-degree type I AV block	Short episodes of supraventricular and non-sustained ventricular tachycardia	Short episodes of supraventricular and non-sustained ventricular tachycardia
Arterial hypertension	+	+	+
Venous pathology of the lower extremities	Deep vein thrombosis after immobilization due to fracture of the left leg	Varicose veins	Varicose veins; thrombophlebitis
Lymphedema	−	+	+
Additional features/comorbidities	Primary arterial hypertension; astigmatism; gynecomastia; abdominal stretch marks; hindfoot deformity; broad foot; joint hypermobility	Primary arterial hypertension; gastric erosions; joint hypermobility; myopia	Resistant arterial hypertension; dyslipidemia; chronic kidney disease Stage 3a; type 2 diabetes; breast cancer (left mastectomy has been performed)

BMI, body mass index; LV, left ventricle; LVEDD, left ventricular end-diastolic diameter; MV, mitral valve; TTE, transthoracic echocardiography; TV, tricuspid valve

The proband was referred to a clinical geneticist for examination because of the suspicion of Marfan syndrome, overgrowth syndrome, or inherited metabolic diseases. According to medical history, the proband had delayed psychomotor development, tall stature, and scoliosis manifesting since early childhood. Sociodemographic factors include learning difficulties and special education in secondary school, no employment history. The proband denied smoking or regular consumption of alcohol. The pectus excavatum was corrected surgically. Deep vein thrombosis was diagnosed at the age of 23 after a leg fracture. Subsequent hematological evaluation did not identify any pathological findings indicative of antiphospholipid syndrome or hereditary predisposition to thrombophilia. Ophthalmological examination confirmed the presence of astigmatism and myopia (− 0.8). At the last evaluation, the proband was 191 cm tall, weighed 120 kg (body mass index (BMI) 33), and had a head circumference of 63 cm. The phenotype was remarkable for coarse facial features, horizontal thick eyebrows, synophrys, malocclusion of teeth, hypertrichosis, gynecomastia, abdominal stretch marks, kyphoscoliosis, knee joint valgus deformity, broad feet, and joint hypermobility. Neuropsychological evaluation revealed mild intellectual disability on the Wechsler Intelligence Scale for Children (version III) (WISC-III) (verbal IQ, 75; non-verbal IQ, 55). The estimate of systemic features was 5 points, according to the revised Ghent criteria for the diagnosis of Marfan syndrome [29], therefore the patient did not meet the clinical diagnostic criteria (< 7 points). Single-nucleotide polymorphism microarray (*SNP* array) analysis of blood DNA showed normal results. Dry blood tests for inherited metabolic diseases (mucopolysaccharidosis, Gauche) showed no abnormalities.

After the initial examination, the proband refrained from consulting a cardiologist for several years due to the absence of symptom exacerbation. During follow-up visit three years later, progression of the aortic root [from 4.8 to 5.3 cm (Z score 6.46)], proximal ascending aorta dilatation [from 4.1 to 6.0 cm (Z score 9.14)] (Fig. 2B) [30], and mitral valve (MV) regurgitation (from moderate to severe) were identified, indicating the need for surgical correction. In addition, mild-to-moderate aortic and mild tricuspid regurgitation were newly diagnosed. Transesophageal echocardiography revealed mitral prolapse and a cleft of the posterior leaflet of the MV (Fig. 2C, D). Repeated 24-h Holter monitoring showed short episodes of non-sustained ventricular tachycardia (up to five QRS complexes) (Fig. 2E) and first- and second-degree type I AV blocks. The proband was offered surgical treatment but was initially reluctant. After extensive consideration, the patient consented to the intervention. However, prior to the scheduled operation, the individual experienced sudden death at home. The relatives refused to consent a pathological examination and the exact cause of death remains undetermined.

Patient 2: The brother of the proband, 39 years of age, had tall stature (195 cm), obesity (weight 140 kg, BMI 38), head circumference 62 cm (90–97‰), milder facial features than the proband, horizontal thick eyebrows, synophrys, abdominal stretch marks, joint hypermobility and skeletal abnormalities (Table 1, affected sibling). Scheuermann’s kyphosis with critical spinal cord compression was surgically corrected at 38 years old. Sociodemographic factors: denied learning disabilities, attended a mainstream school that followed the standard educational curriculum, studied at a college, employment history includes only low-skilled labor positions. The patient denied smoking or regular consumption of alcohol.

The patient complained of chest pain, frequent episodes of palpitations, and dyspnea on exertion. TTE revealed LV dilatation with LVEDD reaching 5.9 cm (indexed 2.22 cm/mm^2^), normal LV systolic function and mild mitral regurgitation. In addition, an aortic root dilatation of 4.5 cm (Z score 2.96) [30] was identified on CT angiography with mild coronary artery stenosis (< 30%). Cardiac MRI revealed a dilated LV and RV (increased LV and RV volumes, LVEDD 5.7 cm) with normal ventricular function, atrial dilatation, no signs of LV non-compaction, and no LGE. A 24-h Holter monitoring revealed short episodes of paroxysmal supraventricular tachycardia, non-sustained ventricular tachycardia, and pauses of up to 2.8 s. Other comorbidities: primary arterial hypertension, history of gastric erosions and myopia.

Patient 3: The mother, 58 years of age, had obesity (130 kg, BMI 45), milder facial features than the proband, horizontal thick eyebrows, lymphedema in the legs, varicose veins in both legs, trophic ulcers in the calves, post-thrombotic syndrome, resistant hypertension, type 2 diabetes mellitus, and skeletal problems such as spinal disk herniation*,* spinal osteochondrosis, and bilateral gonarthrosis (Table 1, mother). Sociodemographic factors attended a mainstream school that followed the standard educational curriculum, and employment history includes only low-skilled labor positions*.* The patient denied smoking or regular consumption of alcohol.

The patient was consulted for dyspnea, chest pain, and heart palpitations. Chest CT angiography, pulmonary ventilation, perfusion scan, and dobutamine echocardiography were performed, but no pathology was identified. TTE revealed LV dilatation (LVEDD—6.7 cm (indexed 2.85 cm/mm^2^) with mild-to-moderate mitral and mild tricuspid regurgitation with normal LV systolic function. Cardiac MRI showed LV dilatation (LVEDD—6.5 cm), increased LV volumes, a non-dilated aortic root and ascending aorta, no signs of LV non-compaction, and no LGE. Short episodes of paroxysmal supraventricular tachycardia and non-sustained ventricular tachycardia were observed during 24-h Holter monitoring. CT coronary angiography revealed mild coronary artery stenosis (< 30%).

### Next-generation sequencing

Exome sequencing was performed on proband genomic DNA using HumanCore Exome Kit (Twist Bioscience), as described previously [31]. Genes associated with cardiovascular abnormalities and intellectual disabilities were analyzed. Variants were classified according to the guidelines of the American College of Medical Genetics and Genomics [32]. Only variants that passed the quality and coverage filters and showed > 99.9% detection reliability were analyzed.

### PCR and Sanger sequencing

PCR primers were designed using the Primer-BLAST tool. For analysis of the cDNA sequence, a forward primer designed for *DNMT3A* exon 18 and a reverse primer spanning the exon 21 and exon 22 junction were used.

DNA samples from the sibling and parents were analyzed by Sanger sequencing for family segregation analysis. To verify the probable impact of the identified variant on the splicing process, cDNA Sanger sequencing was performed for the proband, his brother, and his mother. PCR products were sequenced with the BigDye® Terminator v3.1 Cycle Sequencing Kit (Thermo Fisher Scientific, Austin, TX, USA). Capillary electrophoresis was performed using an ABI3130xl Genetic Analyzer (Thermo Fisher Scientific, Vilnius, Lithuania). Chromatograms were viewed and analyzed using a viewer Chromas 2.6.6 (Technelysium Pty Ltd, Australia).

### Extraction and complementary DNA synthesis

Total RNA was extracted from the peripheral blood samples of the proband, his brother and his mother using Tempus™ Blood RNA Tube and Tempus™ Spin RNA Isolation Kit (Thermo Fisher Scientific, Austin, TX, USA) according to the manufacturers’ protocol. Complementary DNA (cDNA) was synthesized using a high-capacity RNA-to-cDNA kit (Thermo Fisher Scientific, Vilnius, Lithuania), according to the manufacturer’s guidelines.

### Transcriptome analysis

Total RNA was extracted from peripheral whole blood samples of the probands (proband 1 and proband 2), their mother and six healthy individuals (three females and three males) for control samples using the Tempus™ Blood RNA Tubes (Thermo Fisher Scientific) and Tempus™ Spin RNA Isolation Kit (Thermo Fisher Scientific) system according to the manufacturer’s instructions. The quality and quantity of RNA were assessed using a NanoDrop spectrophotometer (Thermo Fisher Scientific). RNA samples were then sent to service provider ‘Novogene’ for library preparation and transcriptome (mRNA) sequencing using Illumina NovaSeq 6000 platform.

Raw transcriptomic data underwent quality assessment with FastQC v0.11.9 [33] and were trimmed for low-quality bases and adapter sequences using Trimmomatic v0.39 [34]. The cleaned reads were aligned to the human genome (GRCh38 release 111 from Ensembl) utilizing STAR v2.7.11a [35]. Gene-level read counts were obtained with featureCounts v2.0.6 [36]. Differential expression analysis was performed using DESeq2 v1.42.0 [37]. The analysis involved comparing Proband 1 and Proband 2 with age-matched male controls and the mother with age-matched female controls. Log2 fold change adjustments were made using the lfcShrink function with the apeglm option in DESeq2. Differentially expressed genes were identified using a threshold of an FDR-adjusted *p* value < 0.1 and a fold change > 1.5. Enrichment analysis for Gene Ontology terms was performed using clusterProfiler v4.10.1 [38]. Input genes for GO analysis included all differentially expressed genes identified above. The enrichment analysis for Gene Ontology (GO) terms was conducted using the enrichGO function within clusterProfiler. Parameters were configured to include all three main GO ontologies: Biological Process, Cellular Component, and Molecular Function, by specifying ‘ont =“ALL”’. The ‘pAdjustMethod’ was configured as ‘BH’ (Benjamini–Hochberg method). The ‘OrgDb’ parameter was defined as ‘org.Hs.eg.db’ to align with the human gene ID ontology dictionary. Default settings were applied for other parameters, except where specified, with ‘minGSSize’ at 10 and ‘maxGSSize’ at 500. GO terms were considered significant at a ‘pvalueCutoff’ of less than 0.05. All statistical analyses were performed using R version 4.3.2.

### cfDNA analysis

Cell-free DNA (cfDNA) was isolated from the peripheral blood sample of Patient 2 using the MagMAX™ Cell-Free DNA Isolation Kit (ThermoFisher Scientific) following the manufacturer’s protocol.

Sequencing library was prepared using the SQK-LSK114 kit (Oxford Nanopore Technologies). Raw sequencing data were generated on an ONT MinION device utilizing the r10.4.1 flow cell. Basecalling was conducted employing the ‘sup’ (super high accuracy) model alongside the 5mC and 5hmC models for modification calling. Adapter trimming occurred concurrently with basecalling. Subsequently, reads were aligned to the latest GRCh38 assembly using minimap2. Reads exceeding 500 base pairs (bp) were filtered out based on observed template length. Additionally, secondary, supplementary, and unmapped reads, along with reads with mapping quality below 20q, were filtered out using samtools. Reads exhibiting soft clipping on either end were removed using a custom script. Methylation/hydroxymethylation analysis was performed using the modkit tool.

Nucleosome size distribution was compared to healthy and lung adenocarcinoma patient samples using data provided by Katsman et al. [39]. Nanopore sequencing data anonymized in the Katsman et al. study were obtained from the Zenodo repository at https://zenodo.org/records/6642503. The dataset included samples from healthy individuals: BC02, BC03, BC04, BC05, HU005.10, HU005.11, HU005.12, and individuals diagnosed with lung adenocarcinoma: BC01, BC08, BC09, BC10, BC11, S1* and TBRS* (*sequencing library was prepared using a ligation sequencing kit without barcodes). Commands (CMDs) utilized for analysis were as follows: Mapping with minimap2 via Dorado: minimap2 -ax map-ont –MD –L. Filtering supplementary, secondary, unmapped, and reads with quality < 20q using samtools: samtools view -b -h -q 20 -F 0 × 904 < input.bam > 

Cell origin deconvolution was performed on samples TBRS, BC02, BC03, BC04, BC05, HU005.10, and HU005.11 to determine the cell type of origin for the cfDNA. Deconvolution was conducted utilizing the methylation atlas developed by Moss et al. Nanopore data were aligned to Illumina CpG loci identifiers and deconvoluted using the methodology outlined in Moss et al. [40]. The resulting deconvoluted data were visualized using custom code, focusing specifically on comparing healthy cfDNA nanopore read origin cell types relevant to the TBRS sample. All other cell types were grouped together as ‘other’ for clarity.

### Computational analysis of DNMT3A protein sequence and structure

Human DNMT3A protein sequence (UniProt AC: Q9Y6K1) and related protein sequences were obtained from the UniProt database [41]. Multiple sequence alignment was generated using Clustal Omega [42, 43], and visualized using JalView [44]. The structure and interaction data for the DNMT3A protein were queried using the PPI3D web server [45, 46]. The interaction interfaces were further analyzed using the VoroContacts server [47]. A structural model of the mutant protein was generated using the UCSF Chimera [48].

## Results

Whole exome sequencing of the proband’s DNA sample revealed a likely pathogenic heterozygous variant of *DNMT3A*, NM_022552.4:c.2324C > A, NP_072046.2:p.(Ser775Tyr). This variant has not been described in the literature previously. The missense c.2324C > A variant has not been recorded in ClinVar [49], HGMD [50], or other databases. This variant was not observed in the gnomAD v4.0.0 dataset [51]. In silico analysis predicted that this variant is probably damaging to the protein structure/function: Sift—pathogenic (0) [52], PolyPhen—possibly pathogenic (0.997) [53], MutT—pathogenic (0.99) [54], AlphaMissense predicts mutation as pathogenic (0.8848) [55]. Segregation analysis of the family revealed that the c.2324C > A variant was inherited from the 58-year-old mother. Additionally, familial genetic alteration was identified in the 39-year-old sibling of the proband (Fig. 2A). No genetic variant was detected in the father.

As the position of the variant was near an acceptor splice site (second nucleotide from the start of exon 20), analysis of the cDNA samples of the affected family members were analyzed by Sanger sequencing. The results of the analysis showed no changes characteristic to altered splicing associated with genetic variant. A heterozygous DNMT3A variant was identified in the cDNA sequence, indicating that both alleles of the gene were transcribed and are present in the mRNA.

At the protein level, computational analysis and alignment of DNMT3A protein sequences from different organisms revealed that the residue Ser775 is in a highly conserved region of the MTase domain (Fig. 1C). Mutation Ser775Tyr introduces a bulky tyrosine residue instead of a small serine (Fig. 1D). In contrast to other known pathogenic DNMT3A mutations, such as Arg688His, Cys710Tyr, Arg771Gln, Tyr735Ser, Arg736His, Asn838Asp, and Arg882His [56, 57], Ser775 has not been observed to participate in any direct inter-molecular interaction. However, the variant is similarly penetrative which led us to conduct a more thorough computational-structural analysis. The mutated site is positioned near the DNMT3L binding interface (Fig. 1D); thus, this substitution might have influenced this heteromeric interaction. Moreover, in some published DNMT3A structures, the residue Ser775 is also near the linker that connects the MTase and regulatory ADD domains [18]. Therefore, the change to a larger tyrosine residue might affect the dynamics of ADD domain movement. There are examples of mutations in other proteins causing long-range effects upon both protein structures [58, 59] and interactions [60, 61]. Such an indirect effect cannot be ruled out in this case, because Ser775 interacts with several surrounding amino acids, and disruption of this network may affect the overall thermodynamic properties of the protein. As it has been shown, DNMT3A mutant protein instability, subsequent ubiquitination and proteasome-mediated degradation may be the cause of function loss in some cases [62]. Altogether, these observations are consistent with the in silico prediction that this mutation is pathogenic (as previously mentioned). However, further experimental research is needed to validate these in silico findings.

**Fig. 3:**
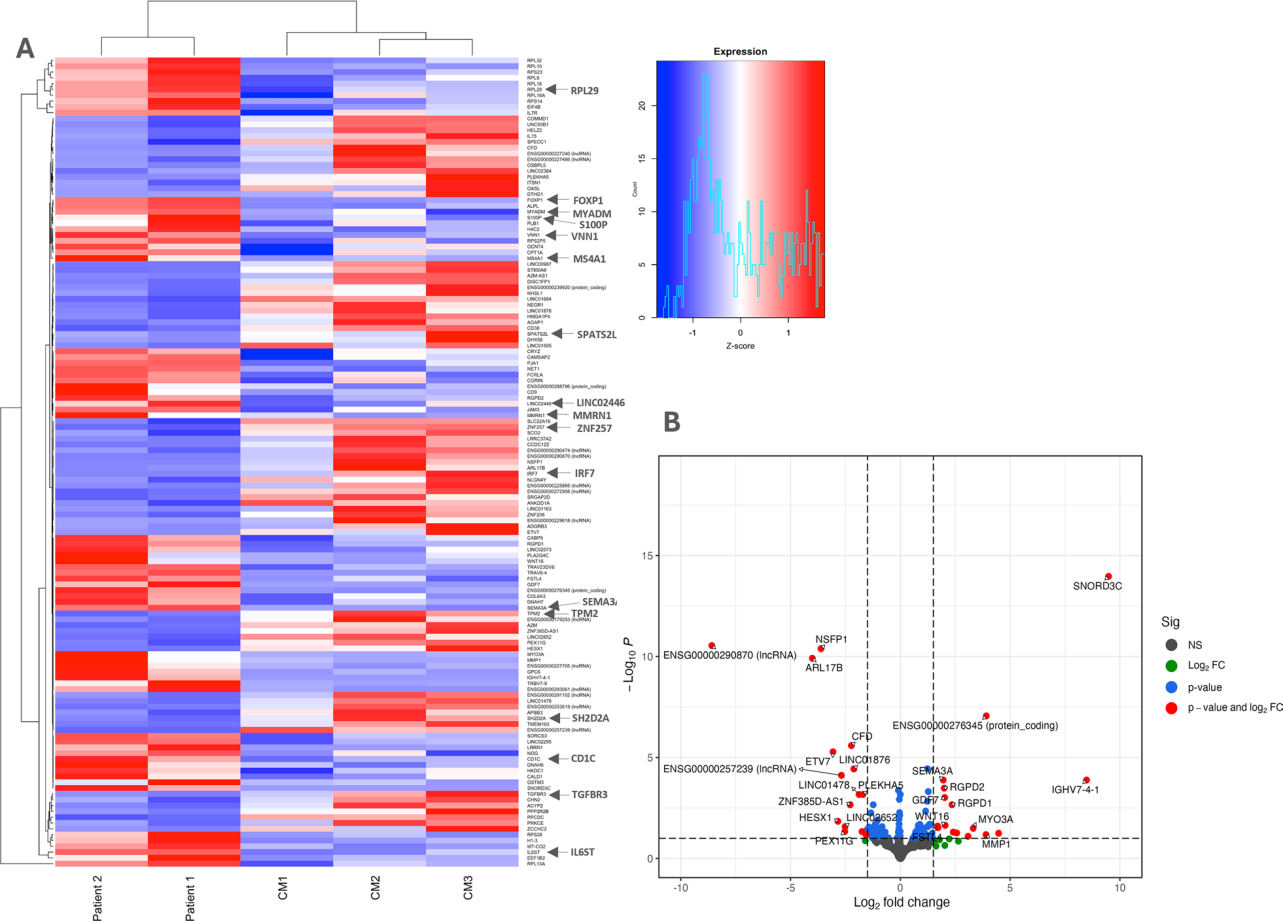
Differentially expressed genes identified in the peripheral blood samples of the probands with a missense variant Ser775Tyr in DNMT3A. **A** Differential gene expression profile in probands with DNMT3A mutation compared to healthy controls. Heatmap represents the expression levels of statistically significant DEGs across two patients harboring DNMT3A mutations (Patient 1 and Patient 2) and three healthy male controls (CM1, CM2, CM3). Each row corresponds to a specific gene, while each column represents an individual subject. Red indicates upregulation, blue indicates downregulation, and the intensity of the color correlates with the magnitude of expression change. Clustering on the left side groups genes with similar expression patterns. **B** Volcano plot of differential gene expression between DNMT3A mutation carriers and healthy controls. The *x*-axis represents the log2 fold change in gene expression, while the *y*-axis shows the negative logarithm (base 10) of the FDR. Genes with significant differential expression (FDR < 0.1) and a fold change greater than 1.5 are depicted above the horizontal dashed line and outside the vertical dashed lines, respectively

To assess gene expression differences, RNA sequencing was performed on both patients and their mother’s peripheral blood samples. As healthy controls, RNA samples of three males of the same age group as the probands and three females of the same age group as the mother were also included for transcriptome sequencing. Differentially expressed genes (DEGs) were identified by comparing the probands (or the mother) with samples from healthy controls with thresholds set to FDR-adjusted P < 0.1 and fold change > 1.5 (Additional File 1: Table S1). As we have two male patients with the same DNMT3A variant and of similar age, we primarily focus on their transcriptome analysis results as more statistically significant. The mother’s data are utilized solely for the purpose of additional comparison of the identified findings (specific genes) within the probands’ data. In total, 139 DEGs were identified of which 69 were upregulated and 70 were downregulated (Fig. 3A, B). Almost 75% of DEGs are protein coding, several long non-coding RNAs (lncRNAs) were identified, as well as several novel transcripts.

Among the most significant and differentially expressed genes was *SNORD3C* (Small Nucleolar RNA, C/D Box 3C) which expression was increased and novel lncRNA gene (ENSG00000290870) with decreased expression (Fig. 3B). No published research was identified linking these DEGs with biological processes relevant to the cardiological phenotypes observed in the affected individuals. Nevertheless, within both the upregulated and downregulated groups of statistically significant DEGs, several genes were found to exhibit associations with the phenotype of interest. As an example, one of them is an upregulated (Log2 Fold change = 0.85 in the male patients, also prominent in the mother’s data) gene *FOXP1* (Forkhead Box P1) (Fig. 3A). The gene is a transcription factor important for the development of various tissues including brain and heart. The cardiac anomalies observed in Foxp1 null mice embryos closely resemble those found in various types of congenital heart disease in humans [63]. In line with our report, Foxp1 gene has been identified as a target for de novo methylation by DNMT3A during mammalian development in mouse embryonic stem cells [64]. Moreover, functional gene analysis using the WebGestalt tool [65] revealed downregulated genes *TPM2* (Log2 Fold change = − 1.3) and *TGFBR3* (Log2 Fold change = − 0.73) which are associated with aortic aneurysms. *TPM2* downregulation shown in analysis of abdominal aortic aneurysm patches has been implicated in the mechanism underlying the disease progression [66]. Our patient 1 had proximal ascending aorta aneurysm, which again reinforces the importance of these genes in aneurysm formation and highlights a possible *DNMT3A* involvement in the regulation of vascular genesis.

Several other DEGs (Fig. 3A) were also found to be associated with cardiac molecular biology and physiology through literature and database analyses. Notable among the upregulated were genes involved in protein synthesis (e.g., *RPL29*), immune system function (e.g., *IL6ST*, *MS4A1*, *CD1C*), and signaling pathways (e.g., *SEMA3A*, *WNT16*) [67–71]. The downregulated genes exhibited associations with transcriptional regulation (e.g., *ZNF257*), signaling pathways (e.g., *SPATS2L*, *S100P*) [72–74]. While this list of genes spans various molecular process categories, research studies have also investigated their roles in elucidating cardiovascular biology. Of note, *DNMT3A* gene expression remained unchanged across all samples.

A subset of the DEGs identified in this study (e.g., *MYADM*, *MMRN1*, *IRF7*, *LINC02446*, *CD9*, *VNN1*, *SH2D2A*) are in line with the published results describing peripheral blood samples from a patient with TBRS caused by *DNMT3A* missense variant Arg882His [16], reinforcing the importance of these genes in TBRS pathogenesis.

**Fig. 4:**
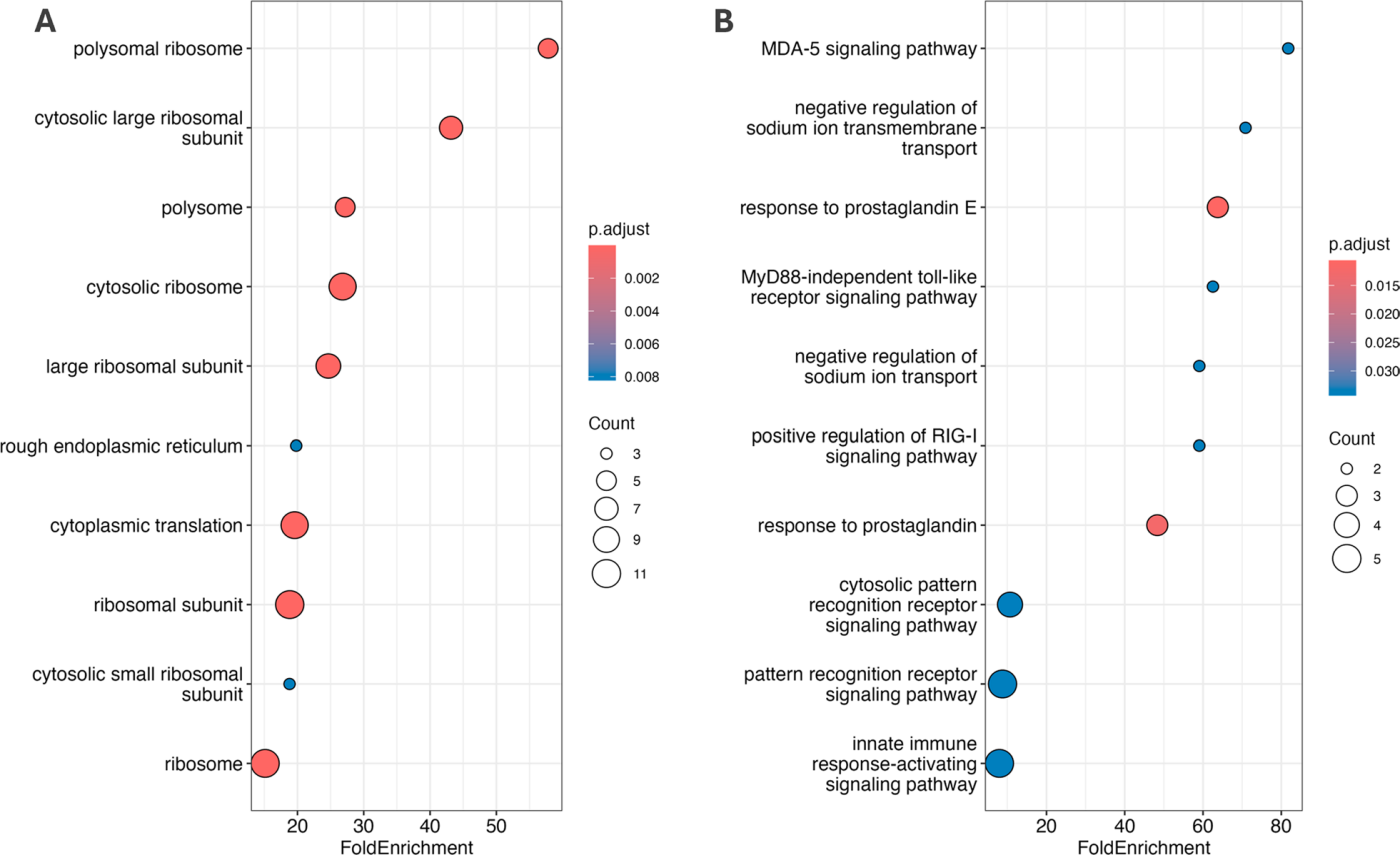
Gene ontology (GO) enrichment analysis of the probands with a missense variant Ser775Tyr in DNMT3A. **A** Dot plot illustrating GO enrichment analysis results for overexpressed genes in individuals with a DNMT3A missense variant. The fold enrichment on the *x*-axis quantifies the relative overrepresentation of these terms in the context of the total genome. The color of the dots indicates the statistical significance after adjustment. **B** Dot plot illustrating GO enrichment analysis results for genes that are underrepresented in the gene set from individuals with a DNMT3A missense variant. The *x*-axis shows the fold enrichment value, contrasting the proportion of specific GO terms in the dataset against their distribution in the reference genome. Dot color signifies the adjusted level of statistical significance

In the gene ontology (GO) enrichment analysis, we identified several significantly enriched biological processes and cellular components associated with the DEGs. In the group of upregulated genes, cellular components including ‘polysomal ribosome’ (*p* < 0.002), ‘cytosolic large ribosomal subunit’ (*p* < 0.002), and ‘polysome’ (*p* < 0.002) were among the top enriched categories (Fig. 4A). In the group of downregulated genes, significantly enriched were biological processes such as ‘MDA-5 signaling pathway’ (*p* > 0.03), ‘negative regulation of sodium ion transmembrane transport’ (*p* > 0.03), and ‘response to prostaglandin E’ (Fig. 4B). While these findings do not unequivocally elucidate the connection between disrupted molecular processes and the phenotypes of affected individuals, they indicate the potential existence of biological pathways and mechanisms that underlie this complex syndrome. Based on literature review, it could be suggested that ribosome protein quantity and translational efficiency play important roles in certain cardiac conditions. Dysregulation of ribosome biogenesis and protein translation can lead to pathological changes in the heart, such as hypertrophy, and understanding their roles in cardiac conditions could offer insights into disease mechanisms and therapeutic strategies [75–77].

**Fig. 5:**
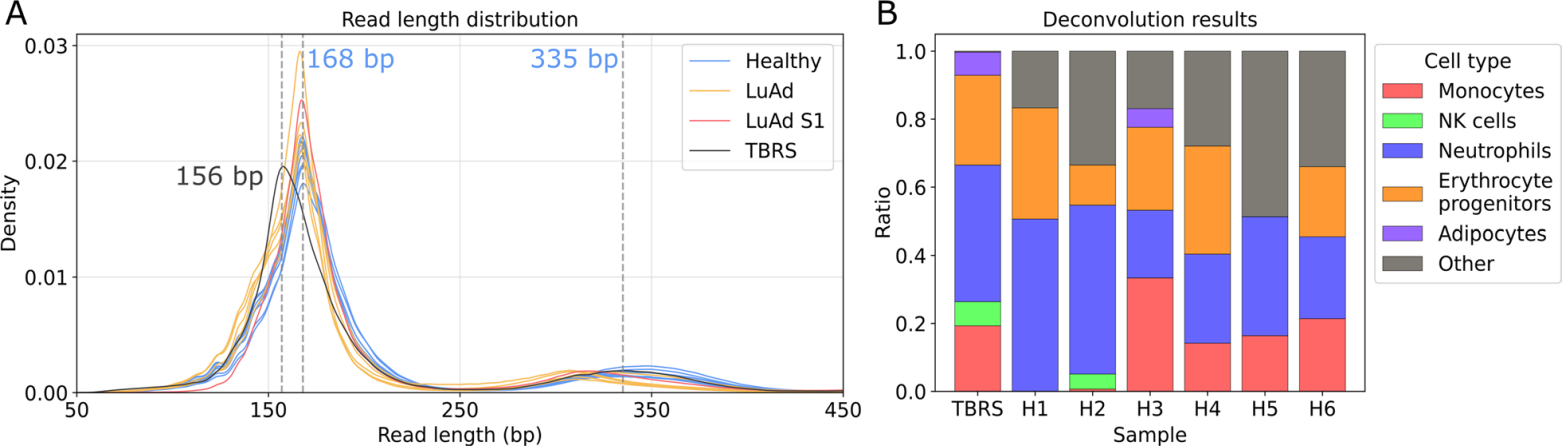
Blood cfDNA of TBRS patient 2 nanopore sequencing and analysis. **A** cfDNA fragment density plot. Fragment size distribution is compared among the TBRS patient, seven samples of healthy individuals and six lung adenocarcinoma patients (LuAd). Sample S1 represents a lung adenocarcinoma patient whose cfDNA library was prepared in the most similar fashion to that of TBRS. **B** Cell origin deconvolution of cfDNA reads using the Moss et al. [40] method to determine the cellular source of cfDNA fragments. TBRS patient results are compared to six healthy (H1-6) individuals from Katsman et al., 2022 study [39]

Cell-free DNA analysis revealed a significant shift in mononucleosome fragment size down to shorter fragments (peak at 156 bp) as compared to healthy control data (peak at 168 bp) (Fig. 5A). Dinucleosome fragments are similar in the TBRS and healthy samples (peak around 335 bp). It has been shown previously that cfDNA from cancer patients exhibit trends toward shorter nucleosome fragments [39] which was attributed to hypomethylation and increased nuclease activity [78, 79]. As hypomethylation at least in some DMRs in TBRS was expected [16], we took nanopore-sequenced lung adenocarcinoma cfDNA samples for comparison [39]. The fragmentation distribution (Fig. 5A) shows a unique pattern for the TBRS sample, as compared to both healthy and cancer samples. Further studies would be of interest, if this kind of analysis could be implemented in diagnostics and research of TBRS.

Nanopore sequencing allows direct methylation (5mC) and hydroxymethylation (5hmC) detection. In our TBRS cfDNA sample global CpG modification was estimated to be: 75.8% 5mC and 2.4% 5hmC, 78.2% in total, which is very similar to the amounts published for non-R882 DNMT3A variant peripheral blood with bisulfite sequencing analysis [62]. Unfortunately, we were not able to perform DMR analysis due to low genome coverage left after all the data filtration processes, which we implemented to assure that only the best quality reads were enrolled in further analyses. Further, we used our data to deconvolute the cells of origin of the cfDNA and compared it to the healthy controls from Katsman et al. [39]. No unique cells were found for the TBRS sample, only monocytes, NK cells, neutrophils, erythrocyte progenitors and adipocytes, all in comparable amounts as in the healthy controls (Fig. 5B). This indicates that although TBRS patients have multiple congenital abnormalities, no specific tissue experiences any profound degradation.

## Discussion

In addition to overgrowth, intellectual disability, and other known features of TBRS, the proband and his affected family members were diagnosed and thoroughly examined for cardiovascular abnormalities that are less frequently described in the literature. A 34-year-old proband presented with LV dilatation, cleft of the posterior leaflet of the MV, ventricular arrhythmias, progressive MV regurgitation, and aortic dilatation. His affected mother and brother had LV dilatation, mild-to-moderate MV regurgitation, supraventricular and ventricular arrhythmias, and the older brother was diagnosed with aortic root dilatation.

Most individuals with TBRS are children; however, approximately 20 adult individuals have now been reported [1, 5, 8, 9]. The most common cardiovascular findings in childhood were congenital heart defects and anomalies (atrial septal defect, patent foramen ovale, ventricular septal defect, and patent ductus arteriosus) and heart valve pathology (MV prolapse and mitral and tricuspid valve regurgitation) [1, 4, 6, 7, 10, 80]. Cardiomyopathy and aortic root dilatation were less frequently detected and usually in adult patients [1, 5, 8, 9]. Only a few individuals with heart rhythm disorders have been described, including a neonate with bradycardia, a young adult with atrioventricular re-entry tachycardia and mitral regurgitation, and a patient with near-fainting episodes due to postural orthostatic tachycardia syndrome [1, 4]. Unfortunately, there is a paucity of data on the patients' clinical records from adolescence. The family underwent a comprehensive clinical examination for the first time in adulthood.

All three family members described here presented with varying degrees of mitral regurgitation. Atrioventricular valve disease has been described in patients with TBRS during childhood and as young adults [1, 4, 10]. Tatton-Brown et al. reported four cases of MV disease: two 21-year-old adults, one 9-year-old child and one 16-year-old teenager with mitral regurgitation [1]. The third patient also had aortic root enlargement. Balci et al. described an 11-year-old patient with TBRS and a dysplastic MV [4]. Hage et al. reported MV prolapse in a 34-year-old African American woman with TBRS and pituitary adenoma [10]. Another case of MV prolapse was reported in a 14-year-old patient with TBRS who remained stable on repeated echocardiography at the ages of 16 and 18 years [5]. To our knowledge, no affected individual has been described with a cleft of the posterior mitral leaflet in TBRS. It is a rare congenital cause of mitral regurgitation and is usually associated with an atrial septal defect, mitral prolapse, papillary muscle malrotation, and Marfan syndrome [81, 82].

Cardiomyopathy has been previously reported in only two individuals, 41-year-old and 34-year-old men with TBRS [5, 9]. For the first individual, cardiomyopathy was not specified in detail [9]. The latter patient revealed dilated cardiomyopathy via cardiac MRI: severe LV dilatation (maximum end-diastolic diameter of 7.0 cm) and reduced LV systolic function to 34% [5]. In our reported family, the proband had the most dilated LV (LVEDD—6.9 cm) with LV non-compaction. His mother had slightly less LV dilatation (LVEDD—6.7 cm on TTE, 6.5 on cardiac MRI). However, all the family members had preserved LV EF.

Aortic root dilatation is another less frequent cardiovascular abnormality in patients with TBRS. Only three cases have been reported in the literature. Tatton-Brown et al. have described aortic root enlargement in a 16-year-old patient who also had MV regurgitation [1]. The other two were adults in their fourth decade [5, 8]. The first was a 30-year-old man with an aortic root dilatation of 5.7 cm and tall stature, who was referred to a genetic clinic for suspected Marfan syndrome. Whole exome sequencing revealed a de novo nonsense variant of DNMT3A, c. 1681G > T (NM_022552.4), and genetic testing for Marfan syndrome was negative. The father of this patient also had aortic root dilatation. As there were no data on cardiac abnormalities in patients with TBRS at that time, the investigators concluded that aortic root dilatation was related to the DNMT3A variant [8]. The second reported adult was a 34-year-old man with TBRS and an aortic root dilatation of 4.4 cm (Z = 2.39–3.17) at the sinuses of Valsalva. This patient also had dilated cardiomyopathy with reduced LV EF to 34%. A DNMT3A de novo variant c.1904G.A (NM_175629.2) was identified [5]. Our proband had dilatation of the ascending aorta that was more prominent than aortic root, with significant progression over time, whereas his brother had an aortic sinus dilatation only. This suggests that in adult patients with TBRS, more attention needs to be focused not only on congenital heart defects and valvular pathology, but also on aortic diameters and their changes over time.

There are limited data on whether TBRS affects lifespan. Sudden death due to TBRS has not been reported previously. As the proband did not undergo a pathological examination, we were not able to ascertain the cause of sudden death; however, cardiac pathology cannot be excluded.

TBRS is typically caused by pathogenic de novo* DNMT3A* variants [1]. Familial TBRS has rarely been described: two individuals with inheritance from a healthy mosaic parent (*DNMT3a* c.2312G > A (p.Arg771Gln) and c.2645G > A (p. Arg882His) missense mutations) and one family with autosomal dominant inheritance [4, 9, 83]. In the latter family, the affected father transmitted the *DNMT3A* c.2323-2A > T splice site mutation to his son and daughter. The TBRS had variable expressivity in the family, with the proband (son) phenotype being more severe than that of the other two affected family members [83]. Similarly, in the family described, the proband exhibited the most severe phenotype. Hereditary cases with a TBRS cardiovascular phenotype have not been described previously. Mosaicism was not identified in our family. TBRS is characterized by variable expressivity even in the affected members of the same family.

The clinical expression of TBRS may depend on changes in protein structure. DNMT3A is a complex protein. Its activity is regulated through autoregulation and multiple interactions with other proteins. Moreover, as a key epigenetic regulator in cells, the enzyme itself affects the activity of many other genes. Altogether, this explains the wide range of clinical features caused by pathogenic variants of *DNMT3A* and their variable expressivity. Moreover, different amino acid substitutions in different parts of the protein may have significantly different effects on the pathways of function loss or dysregulation. Interestingly, some mutations in the DNMT3A PWWP domain cause microcephalic dwarfism as opposed to overgrowth and TBRS [84]. Similarly, different mutations causing TBRS could have their specific effects, based on whether the catalytic function is disturbed directly or by distant substitution effects through altered protein stability, etc., as it has been shown for the R882 and other variants [16]. It has been shown that genome methylation is reduced in TBRS and may be rescued by pharmacological approaches in cases of protein stability loss [16, 62]. However, deciphering the molecular pathology pathways that lead from the mutations to the phenotypic manifestations in TBRS is challenging, and detailed studies of this kind are needed. Nevertheless, with currently increasing sequencing and computational protein analysis capacities, personalized treatments, based on the specific *DNMT3A* variants and the different pathways of their function loss, can be envisioned in the future.

It has been proposed earlier that analysis of epigenetic markers in peripheral blood may reflect cardiovascular disease [85]. Our respective transcriptome analysis revealed a number of DEGs associated with TBRS and the DNMT3A Ser775Tyr mutation in particular. Some of them overlap with already published results on TBRS as well as cardiovascular diseases. Others, including some lncRNAs, are of yet undescribed function and pave new ways for discoveries in the field of epigenetics, DNA methylation and maybe cardiovascular development and disease. Our cfDNA analysis showed a unique nucleosome fragmentation pattern in the TBRS sample, which may be attributed to hypomethylation in some regions [16] and reflect chromatin structure alterations, which would be also interesting to investigate further. Altogether, our findings highlight the importance of epigenetic regulation by DNMT3A on cardiovascular system development and function.

## Conclusions

Analysis of protein structure suggests that the novel missense *DNMT3A* variant NM_022552.4: c.2324C > A, NP_072046.2:p.(Ser775Tyr) is expected to significantly impact the structure and function of the mutated protein. This is in accordance with the in silico predictions of this variant as being pathogenic and the characteristic TBRS phenotype observed in the affected individuals. Further studies are needed to elucidate the exact effects of Ser775Tyr substitution to the structure, interactions, and enzymatic activity of DNMT3A.

Clinical examination of the affected individuals in the family showed variable expression and incomplete penetrance of the specific clinical features of TBRS, including aortic disease and cardiomyopathy that may be major complications of the syndrome. Therefore, patients with TBRS require close follow-up and specific therapeutic management.

### Supplementary Information

Supplementary Material 1: Table S1. Differentially expressed genes list.

## Data Availability

The data that support the findings of this report are available from the corresponding author upon reasonable request. Patient 1: Decipher 524710.
